# Retraction Note: Pre-treatment DWI as a predictor of overall survival in locally advanced pancreatic cancer treated with Cyberknife radiotherapy and sequential S-1 therapy

**DOI:** 10.1186/s40644-018-0164-6

**Published:** 2018-09-04

**Authors:** Yu Zhang, Xiaofei Zhu, Denghui Liu, Jiaqi Song, Huojun Zhang, Jianping Lu

**Affiliations:** 1 Department of Radiology, Changhai Hospital Affiliated to the Second Military Medical University, Changhai Road 168, Yangpu district, Shanghai, 200433 People’s Republic of China; 2 Department of Oncology Radiation, Changhai Hospital Affiliated to the Second Military Medical University, Changhai Road 168, Yangpu district, Shanghai, 200433 People’s Republic of China; 3 Department of Orthopedics, No. 113 Hospital of People’s Liberation Army, East Zhongshan Road 377, Jiangdong District, Ningbo, 315000 People’s Republic of China; 4 Department of health statistics, Second Military Medical University, Xiangyin Road 800, Yangpu district, Shanghai, 200433 People’s Republic of China

## Retraction note

The authors have retracted this article because Figures. 2, 3 and 4, as well as parts of the text have been previously published [1]. This article is therefore redundant. Yu Zhang, Denghui Liu, Jiaqi Song, Huojun Zhang and Jianping Lu agree to this retraction. Xiaofei Zhu has not responded to any correspondence from the publisher about this retraction.
